# Portable Microfluidic Integrated Plasmonic Platform for Pathogen Detection

**DOI:** 10.1038/srep09152

**Published:** 2015-03-24

**Authors:** Onur Tokel, Umit Hakan Yildiz, Fatih Inci, Naside Gozde Durmus, Okan Oner Ekiz, Burak Turker, Can Cetin, Shruthi Rao, Kaushik Sridhar, Nalini Natarajan, Hadi Shafiee, Aykutlu Dana, Utkan Demirci

**Affiliations:** 1 Demirci Bio-Acoustic-MEMS in Medicine (BAMM) Laboratory, Center for Biomedical Engineering, Department of Medicine, Brigham and Women's Hospital, Harvard Medical School, Boston, MA, USA; 2 Demirci Bio-Acoustic-MEMS in Medicine (BAMM) Laboratory, Stanford University School of Medicine, Canary Center at Stanford for Cancer Early Detection, Palo Alto, CA, USA; 3 Department of Biochemistry, Stanford School of Medicine, Stanford, CA, USA; 4 Stanford Genome Technology Center, Stanford University, Palo Alto, CA, USA; 5 UNAM Institute of Materials Science and Nanotechnology, Bilkent University, 06800 Ankara, Turkey

## Abstract

Timely detection of infectious agents is critical in early diagnosis and treatment of infectious diseases. Conventional pathogen detection methods, such as enzyme linked immunosorbent assay (ELISA), culturing or polymerase chain reaction (PCR) require long assay times, and complex and expensive instruments, which are not adaptable to point-of-care (POC) needs at resource-constrained as well as primary care settings. Therefore, there is an unmet need to develop simple, rapid, and accurate methods for detection of pathogens at the POC. Here, we present a portable, multiplex, inexpensive microfluidic-integrated surface plasmon resonance (SPR) platform that detects and quantifies bacteria, *i.e.*, *Escherichia coli* (*E. coli*) and Staphylococcus aureus (*S. aureus*) rapidly. The platform presented reliable capture and detection of *E. coli* at concentrations ranging from ~10^5^ to 3.2 × 10^7^ CFUs/mL in phosphate buffered saline (PBS) and peritoneal dialysis (PD) fluid. The multiplexing and specificity capability of the platform was also tested with *S. aureus* samples. The presented platform technology could potentially be applicable to capture and detect other pathogens at the POC and primary care settings.

Emerging micro- and nano-scale bioengineering and biomedical technologies have provided broad applications (*e.g.*, medical diagnostics and biosensors) in health sciences. Developing such platforms that are affordable and rapid for infectious diseases is one of the top priorities for improving human health at the point-of-care (POC) settings^1^,^2^,^3^,^4^. Currently, the standard testing for pathogen detection and quantification are based on cell culture methods, which take 48 to 72 hours^5^,^6^,^7^. Other detection methods such as polymerase chain reaction (PCR) and enzyme linked immunosorbent assay (ELISA), have been widely used to detect and quantify pathogens with high sensitivity and specificity^8^. However, they require expensive equipment and well-trained operators. Additionally, these assays are technically complex and need labor-intensive processing steps. Thus, rapid and inexpensive diagnostic methods are needed that will eliminate peripheral instrumentation and allow to deploy them to the POC. With the ongoing miniaturization in electronics, emerging technologies could allow portable instruments and minimize the need for bulky laboratory infrastructure at the POC and primary care settings^9^. Such self-contained and robust diagnostic devices could also lead to developing strategies for disease monitoring and management^10^.

Microfluidics, being at the convergence of micro/nanoscale engineering, materials science, and biology enables medical solutions for infectious disease diagnostics and monitoring^11^. Microfluidic technologies have been used to manipulate microliter sample volumes and minimize reagent costs in several applications including cryobiology, genetic and proteomic analysis controlling cancer microenvironment, and cell capture and cell release studies^11^,^12^,^13^,^14^,^15^,^16^,^17^. In particular, analysis of bioagents (*e.g.*, pathogens and infectious agents) is possible with microfluidic technologies. For instance, capture and detection of intact viruses on microchips have been demonstrated^18^. Microfluidic based diagnostic technologies have various characteristics, such as inexpensive fabrication, adaptability and rapid results^19^,^20^,^21^. Integrating microfluidic platforms with optical imaging systems combines the advantages of lab-chip platforms with the benefits of optical technologies^22^,^23^. In particular, photonics and plasmonics, *e.g*., surface plasmon resonance (SPR), localized surface plasmon resonance (LSPR) and nanostructured photonic crystals, being at the intersection of nanotechnology and optics, can be used for developing reliable, accurate, easy-to-use biosensor platforms^24^,^25^,^26^,^27^,^28^. Plasmonic lab-chip devices could be constructed as affordable platforms by utilizing single-use, disposable microchips for POC testing. In addition, disease specificity could be attained by using various surface functionalization techniques. In plasmonic sensor technologies, for instance SPR platforms, a biomolecular recognition element is immobilized on metal surfaces (*e.g*., gold, silver) for efficient, specific and selective capture of bioagents. The limit of detection of SPR-based detection systems are affected by factors including the target immobilization method (by affecting the affinity), sample volume and transport properties (by affecting the probability of capturing the target), the refractive index of the target, and practical device parameters^25^,^29^,^30^. Microfluidics helps to handle small sample volumes that affects overall sensor performance and capture kinetics^31^.

Here, we present such a microfluidic-based SPR technology. We validated this portable platform with *Escherichia coli* (*E. coli*) and *Staphylococcus aureus* (*S. aureus*) spiked samples as a model pathogen detection system. Disposable microfluidic chips with gold coated surfaces were functionalized with antibodies for efficient, selective and specific capture of *E. coli* and *S. aureus*. We quantified the captured *E. coli* with brightfield and fluorescence imaging, and analyzed the capture distribution spatially along the microchannels. Limit of detection of the platform was evaluated, and standard curves were generated for *E. coli* spiked in phosphate buffered saline (PBS) and peritoneal dialysis (PD) fluid. Multiplexing and selectivity capability was also assessed with *S. aureus* spiked in PBS samples.

## Methods

### Design and fabrication of microfluidic chips

**Figure 1:**
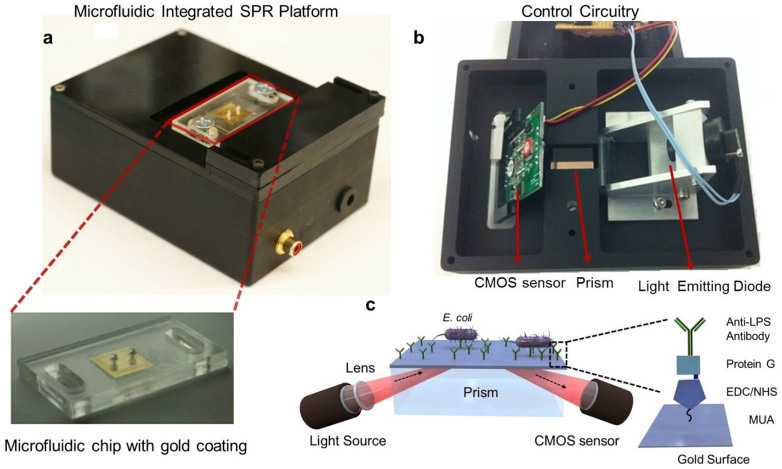
Portable plasmonic platform for pathogen detection and quantification. (a) The surface activated disposable microfluidic chips were mounted on the top side of the device. The microchip with the inlet and outlet ports, and the 50 nm thick gold coated glass substrate along with the disposable microchip is shown below. (b) The electronic setup of the device is represented from bottom. A light emitting diode illuminates a cylindrical lens, which collimates the light onto a rectangular prism. The reflected light is captured by a CMOS sensor, and the image is transferred to a portable computer using the control circuitry. The microfluidic chip is placed on the rectangular prism, with an refractive index matching oil in between. (c) Schematics of the microfluidic integrated SPR platform. The gold surfaces were modified with several activators (*i.e.*, 11-Mercaptoundeconoic Acid (MUA), N - (3 - Dimethylaminopropyl) – N′ -ethylcarbodiimide Hydrochloride (EDC), N-Hydroxysuccinimide (NHS) and with anti- Lipopolysaccharide (LPS) antibody to capture the *E. coli*. The bacteria are captured by the antibodies in the microchannel, and the capture event induces a change in the local refractive index. This change provides a signature on the reflected light, which is captured by the sensor and transferred to a computer for analysis.

The microfluidic chip design comprises a single microchannel with an inlet and an outlet port. The microchip with dimensions 31 mm × 57 mm × 7 mm was constructed as a cartridge for the platform. Two PMMA (poly methyl methacrylate) (3.0 mm thick; McMaster Carr, Atlanta, GA) layers were assembled using a layer of double sided adhesive (DSA, 50 μm thick; iTapestore, Scotch Plains, NJ). A second DSA layer (50 μm thick) and a gold coated substrate formed the microchannel. The microchannel (12 mm × 7 mm × 50 μm) was located in the center of the microchip. The PMMA-DSA-PMMA-DSA-gold chip was assembled as a single use, disposable microchip (Figure 1 and S1). To fabricate the chip, the PMMA and DSA were cut using a laser cutter (Versa Laser™, Scottsdale, AZ). The two PMMA layers were assembled with a layer of DSA. Two openings were cut on the PMMA layer (0.7 mm diameter) that formed the inlet and outlet ports. The distance between these ports was 9 mm. The port openings with diameters of 1.4 mm in DSA allowed fluid transfer without interruption. A second DSA layer formed a microchannel in the center of the microchip with a channel volume of 4 μL. The design of the microchannel included sharp-edged ends. Finally, a gold chip of dimensions 1.4 cm × 1.4 cm was mounted onto the microchip. The microchip design allows future extension of functionality, for instance by incorporating a filter to isolate cells, such as white or red blood cells as shown before^32^.

### Design and fabrication of gold coated glass surfaces

To realize disposable microfluidic chips, glass wafers (Borofloat, Double Side Polished, diameter = 100 mm, t = 0.5 mm) were purchased from University Wafer, Boston, MA (Item 517), and were cleaned with acetone and isopropyl alcohol on the spinner cleaning instrument (Headway PWM-32 Spinner). Then, the wafers were loaded on the sample holders of an electron beam depositor (Denton E-beam Evaporator) for metal deposition. The system was operated at 10^−7^ Torr, and the wafers were deposited with 5 nm of titanium, followed by a 50 nm deposition of gold on a single side. Subsequently, the metal coated wafers were spin coated with a ~ 0.5 μm layer of S1805 photoresist (Shipley 1800-series photoresist) to protect the surface of the gold layer from environmental effects. The spinner was run at 4000 rpm for 40 seconds, and later, baked at 115°C on a hot-plate for 2 minutes. The wafers were cut in 1.4 cm × 1.4 cm square chips using a mechanical dicer (DISCO DAD321 Dicing Saw) and stored after cleaning with distilled water. Before microfluidic chip fabrication, the gold chips were cleaned with solvents to remove any organic residues from the fabrication process. In a solvent bench, chips were placed in an acetone bath and sonicated for 5 minutes. Then, they were transferred to a methanol bath and sonicated for 5 minutes again. Finally, chips were transferred to an isopropanol bath and gently shaken for final cleaning. The gold chips were then dried with nitrogen gas to be used in the fabrication of the microfluidic chips.

### *E. coli* culture and quantification

To analyze and visualize the bacteria distribution on the microchip, a green fluorescent protein expressing plasmid, pRSET/EmGFP (Invitrogen, V353-20), was transferred into the competent *E. coli* strain BL21 Star™ (Invitrogen, C6000-03). According to the manufacturer's instructions, the pRSET/EmGFP plasmid, which confers ampicillin resistance, was incubated at 42°C for 30 seconds with the competent cells. Cells were allowed to cool down on ice for 2 minutes. 250 μL of Super Optimal broth with catabolite repression medium (Sigma-Aldrich, S1797) was added to be incubated for an hour at 37°C, while shaking at 250 rpm. Subsequently, the genetically modified bacteria were spread onto Luria Bertani (LB) agar plates, which contained 100 μg/mL of ampicillin. The plates were incubated at 37°C for 16 hours, and cells were allowed to grow. When colonies appeared, an individually isolated colony was inoculated in a 30 mL of LB broth, containing 100 μg/mL ampicillin. Then, *E. coli* culture was incubated for 16 hours in a 250 rpm rotating incubator at 37°C and aliquoted for later use as a standard stock.

The *E. coli* stock concentration was quantified by diluting the stock solution nine-fold in PBS and spreading the dilution onto LB-ampicillin plates to be incubated at 37°C overnight. Finally, individual *E. coli* colonies were counted, and the concentration of the stock solution was calculated as 10^9^ CFUs/mL. By diluting the stock concentration into PBS, the concentrations in the experiments were obtained.

For peritoneal dialysis (PD) fluid experiments, *E. coli* was spiked in a commercial dialysate, received from Chronic Ambulatory Peritoneal Dialysis (CAPD) Unit from Faulkners Hospital (Baxter Inc., 5B9766). Sample concentrations ranging from 10^5^ to 3 × 10^7^ CFUs/mL were prepared by serial dilution in PD fluid.

### *S. aureus* culture and quantification

*S. aureus* (ATCC #25923, American Type Culture Collection, Mannassas, VA) cells were hydrated and streaked for isolation on a Luria Bertani agar (LA) plate. Following growth, a single isolated colony was selected and inoculated in 3 mL of Luria bertani (LB) media. The bacteria culture was grown on an incubator shaker for 18 hours at 37°C, 250 rpm until it reached the stationary phase. Quantification of *S. aureus* stock concentration was done by diluting the overnight cultures nine-fold in PBS. Diluted cultures were streaked onto LB Agar plates, incubated at 37°C overnight and individual *S. aureus* colonies were counted after overnight incubation. The concentration of stock cultures was calculated as 10^9^ CFUs/mL. For validation experiments, the overnight cultures were diluted at a ratio of 1:10 in LB media.

### Portable biosensor operating principle and fabrication

The SPR platform was custom-made for microfluidic integration (Figure 1a, b). The design of the system was based on Kretschmann configuration, which uses prism coupling to satisfy momentum conservation for plasmon excitation by an external light source^33^ (Figure 1c). A collimated point source light emitting diode (LED) output (λ = 705 nm) was focused with a cylindrical lens (f = 15 mm) and passed through a glass prism (N-BK7, n = 1.51) to illuminate the surface of the microchip. The rectangular prism was positioned on a stage, which was practical for inserting microfluidic chips and the prism. Reflected light was captured by a CMOS sensor (500 × 582), which was placed such that the normal of the sensor surface was parallel to the monitored light direction. The light source, CMOS sensor, and the associated optical and electrical components were packaged in a portable box with dimensions of 13.5 cm × 10 cm × 5.2 cm (length × width × height) (Figure 1a, b). The total weight of the packaged system was 0.85 kg (1.87 lbs). The disposable microfluidic chips were then placed on the prism with a thin layer of index matching liquid (n = 1.5000 ± 0.0002, Series A, Cargille Labs, NJ) and fixed in place (Figure 1a). The index matching liquid was used for providing lossless optical transmission between the prism and the glass substrate on the microchip. A custom-designed software was used to monitor the resonance angle changes. The software captured the image frames from the sensor, calculated the resonance angle in real time, and then, plotted the resonance curve and the sensogram (resonance angle as a function of time) as a readout for kinetic measurements.

### Calibration

To calibrate the sensor, fluids (i.e., distilled water PBS and ethanol (200 proof)) with known refractive indices were used as reference samples, and their plasmon resonance curves were measured in microfluidic channels. Then, the instrument parameters were matched to these experimental curves to extract device fitting parameters. In the Kretschmann configuration, the surface plasmon mode's wave vector, k_sp_, is described by 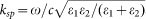, where *ω* is the angular frequency of the incoming light, c is the speed of light, ε_1_ and ε_2_ are the dielectric permittivity of the gold and biosensor medium^34^. The resonance condition occurs when the surface plasmon mode wave vector is equal to the incident light's wave vector in the plane of the interface, i.e., k_sp_ = *ω*/c (*n* Sinθ) where *n* is the refractive index of the prism and θ is the incidence angle of the light^35^. The calibration procedure allowed an active operating range of 67 to 73 degrees for the resonance angle and the smallest angle shift the device showed sensitivity was 0.002°. All calibration measurements and validation experiments were performed at room temperature.

### Functionalization of microchannels for label-free *E. coli* and *S. aureus* capture and detection

The surface modification for *E. coli* and *S. aureus* detection was performed on the 50 nm gold coated glass wafers. The gold surfaces were first submerged in acetone bath at 56°C to clean any organic residues from the nanofabrication and chip dicing. The gold chips were incubated with 10 mM of 11-Mercaptoundecanoicacid (MUA) (450561 Aldrich – Sigma Aldrich) dissolved in ethanol overnight, and thus, self-assembled monolayer with carboxyl groups was generated onto the surface. After MUA modification, microfluidic channels were constructed. Then, a 100 μL of, 1:1 mixture of 100 mM N- (3 - Dimethylaminopropyl) – N′ -ethylcarbodiimide Hydrochloride (EDC, 03450 Fluka – Sigma Aldrich) in 10 mL MES (M3671 Sigma – Sigma Aldrich) buffer (9,76 μg/mL in dH_2_O) and 50 mM of N-Hydroxysuccinimide (NHS, 130672 Aldrich – Sigma Aldrich) in 10 mL of MES buffer, was loaded with pipettes to microchips through the inlet port and incubated for 30 minutes. EDC reacts with carboxyl groups for the formation of amine reactive intermediate that is stabilized by the addition of NHS. The final chemical product is a succinimide group that reacts with amine groups of organic compounds (*e.g.*, proteins). The surfaces were then washed with 100 μL of distilled water and 300 μL of PBS. This step was followed by introducing 100 μL of Protein G (0.1 mg/mL) (21193 – Thermo Scientific) dissolved in PBS for the immobilization of antibodies with their favorable orientations, and Protein G was incubated at 4°C for an hour. The surfaces were then washed with 300 μL of PBS. 100 μL of anti-lipopolysaccharide (LPS) antibody (ab35654 – Abcam) (5 μg/mL) solution in PBS was incubated for 30 minutes, and thus, antibodies were immobilized on the Protein G coated layer to capture GFP-expressing *E. coli.* For *S. aureus* experiments, 100 μL of anti-lipotheichoic acid (anti-LTA) antibody (MA1-7401 – Thermo Scientific) (5 μg/mL) solution in PBS was incubated on Protein G-coated surfaces for 30 minutes. After another wash with 300 μL of PBS, various concentrations of *E. coli* ranging from 3.2 × 10^5^ to 3.2 × 10^7^ CFUs/mL in PBS were passed through the chip at 5 μL/min rate for detection. In PD fluid experiments, 10^5^ to 3.2 × 10^7^ CFUs/mL of *E. coli* spiked in PD fluid were passed through the chip at 5 μL/min rate for detection. In *S. aureus* experiments, 5 × 10^6^ CFUs/mL were tested for multiplexing and selectivity of the platform.

### Statistical Analysis

To evaluate each SPR angle shift, we facilitated one-way analysis of variance (ANOVA) with Tukey's *posthoc* test followed with Bonferroni's Multiple Comparison Test for equal variances for multiple comparisons with statistical significance threshold set at 0.05 (p < 0.05). Error bars in the plots represented standard error of the mean (SEM). All statistical analyses was performed using GraphPad Prism 5 software (GraphPad Software, Inc., La Jolla, CA).

## Results and Discussion

### *E. coli* capture and quantification in the microchannels

**Figure 2:**
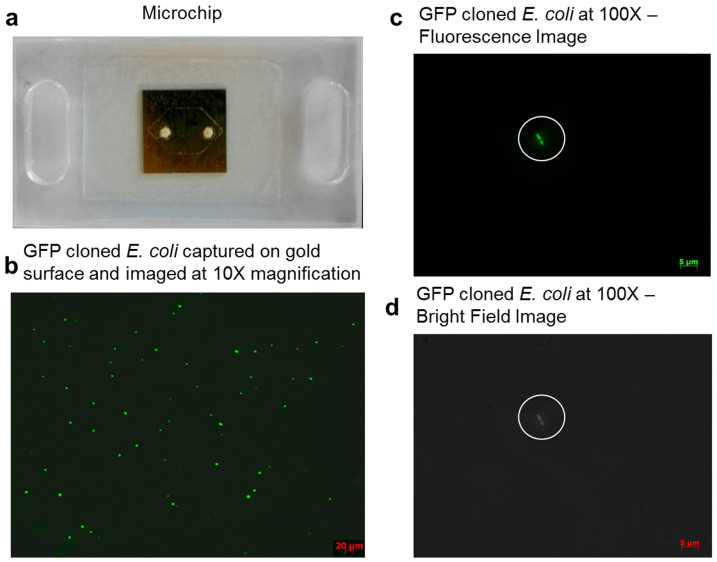
Microfluidic chip and fluorescent images of *E. coli* taken under 10× and 100× magnifications in microchannels. (a) Microfluidic chip and the geometry of the channel formed by the 50 μm thick double sided adhesive tape. (b) Captured GFP-expressing *E. coli* on the gold surface was fluorescently imaged at 10× magnification in the centre of the microchip. 75 μL of 10^6^ CFUs/mL *E. coli* in PBS was run through the chip. The 10× fluorescence image was taken in the centre of the microchannel and was used to count the green fluorescing spots to give a count of 78. (c) Captured GFP-expressing *E. coli* was fluorescently imaged at 100× magnification. Bacteria are indicated with white circles. (d) Bright-field image at 100× magnification shows the morphology of bacteria, indicated with white circles, for the same image shown in (c).

To capture *E. coli* on microfluidic chips, we utilized a Protein G-based surface chemistry that allows to immobilize antibodies in a favorable orientation^18^. It was previously shown that anti-LPS presented highest capture efficiency among a set of antibodies (antiflagellin, anti-LPS and CD14) for on-chip *E. coli* capture when Protein G based surface chemistry was performed^6^. We adapted the surface chemistry for gold-coated substrates, to target *E. coli* capture on a microfluidic chip. The captured bacteria counts between the inlet and outlet ports were evaluated, for chip characterization as well as for the limit of detection measurements. To quantify the number of bacteria captured on gold surfaces, a 75 μL of 10^6^ CFUs/mL *E. coli* was passed through surface functionalized microchips (Figure 2a) at a 5 μL/minute flow rate using a syringe pump. The GFP-expressing *E. coli* was fluorescence imaged under 10× magnification (Figure 2b). To evaluate the capture specificity, brigthfield images and fluorescence images at 100× magnification of the same spot were compared (Figure 2c, d).

We first addressed two considerations, *i.e*., autofluorescence and clustering, which could potentially result in erroneous estimates of bacterial counts. Autofluorescence may interfere with the samples under study and could potentially result in erroneous estimates of bacterial counts. To confirm that the fluorescence spots in the images were coming from the bacteria (not from autofluorescence of other particles), we gathered bright field images of 16 locations (99 μm × 66 μm) on a chip at 100× magnification and compared the morphology of *E. coli* observed in these images with green spots observed in the corresponding fluorescence images under 100× magnification. These images showed no autofluorescence effects (Figure 2c, d). Additionally, clustering of bacteria could also potentially result in under-quantifying the bacteria on the sample surface. Therefore, we required that each observed point with fluorescence under the 10× magnification images should be associated with a single bacteria and not resulting from the clustering of *E. coli*. To confirm these observations, we took a fluorescence image under 10× magnification and 25 fluorescence images with 100× magnification uniformly sampling the 10× image area. Using the twenty-five 100× images, we estimated a total of 82 *E. coli* on the imaged surface under 10× magnification. In comparison, 78 fluorescing points were counted in the 10× image. We concluded that clustering did not also play a significant role in the 10× image *E. coli* counts. Therefore, in the consecutive capture distribution analysis and limit of detection experiments, fluorescent images with 10× magnification are utilized for bacterial counts.

### Capture Distribution Experiments

**Figure 3:**
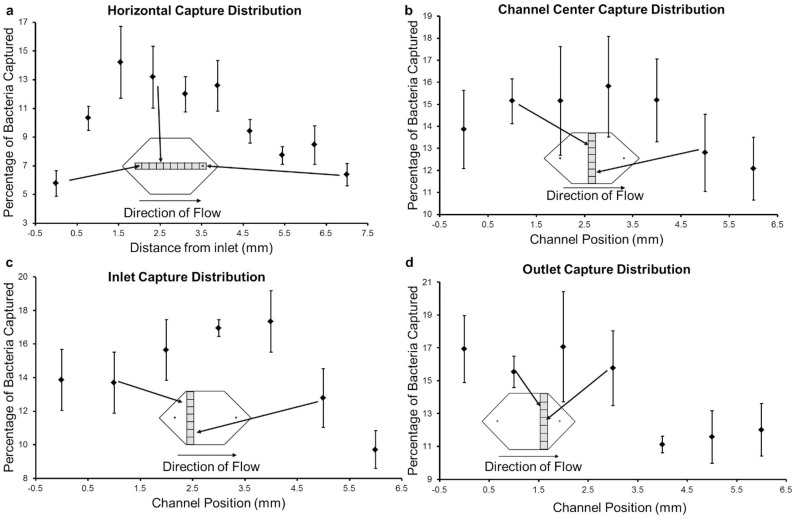
Capture distribution of *E. coli* in microchannels. (a) The plot represents the overall percentage of captured *E. coli* in the microchannels through a horizontal stripe passing from the inlet port to the outlet port. The error bars represent the standard error of the mean (n = 6). The capture distribution spatially peaks at 2 mm from the inlet port. During each run, 100 μL of 10^6^ CFUs/mL *E. coli* was passed through the channel. (b) Three stripes perpendicular to the channel direction were evaluated for capture distribution for the same chips evaluated in (a). The capture distribution for the stripe passing through the center of the channel is shown. (c) The distribution for the stripe passing close to the inlet port is shown. (d) The capture distribution for the stripe passing close to the outlet port is shown. The error bars indicate standard error of the mean normalized to the total *E. coli* count on each chip (n = 6). Approximately a uniform capture distribution was observed in the direction perpendicular to the flow.

To evaluate the capture distribution of bacteria, six microchips were tested with 100 μL of 10^6^ CFUs/mL *E. coli* spiked in PBS. The cell capture distribution along the microchannels were evaluated by identifying one longitudinal region passing through the inlet and outlet ports and three additional regions that are perpendicular to the flow direction in the microchannel. The captured *E. coli* were then manually quantified using fluorescence images taken under 10× magnification. The longitudinal region passing through the ports and center of the microchannel was evaluated with 10 images (0.987 mm × 0.658 mm) under 10× magnification (Figure 3a). The evaluated region covered the distance between the inlet and outlet ports. The maximum capture of *E. coli* was observed at 2–3 mm away from the inlet port and was consistent with previous microfluidic experiments on glass substrates^36^. The three regions perpendicular to the flow direction were also evaluated with fluorescence images under 10× magnification. One region perpendicular to the flow and passing through 1 mm to the right of the inlet, a second one passing through 1 mm to the left of the outlet and a third one passing through the center of the chip were evaluated with 7 images (0.987 mm × 0.658 mm) each (Figure 3b, c, and d). The capture distribution showed a slight peak around the center of these regions but in general a uniform distribution was observed in the direction perpendicular to the flow.

### SPR angle and kinetic measurements

The field of view of the sensor corresponded to an area of 1 cm × 0.5 cm on the gold chip surface (Figure S2). The resonance angle was defined as the angle at which there was maximum light coupling to the plasmon modes^37^. The resonance condition was satisfied at angles corresponding to a band which was observed as a dark shadow on the camera (Figure S2a). The custom-developed software averaged the pixel intensities in a selected viewing area and converted the mean intensity as a function of distance to mean intensity as a function of the incidence angle. The minimum point of this band was calculated in real time and the SPR sensogram was generated, allowed the probing of surface events. A two inlet microchip was prepared for evaluating the rise time (*i.e.*, time to increase from 10% to 90% of the resonance angle difference between the water and PBS signals) of the platform. Distilled water and PBS were alternately applied by syringe pumps at 5 μL/min flow rate, and the switching of liquids was evaluated. The rise time of the switching was observed as 8 seconds (Figure S2b, S2c).

**Figure 4:**
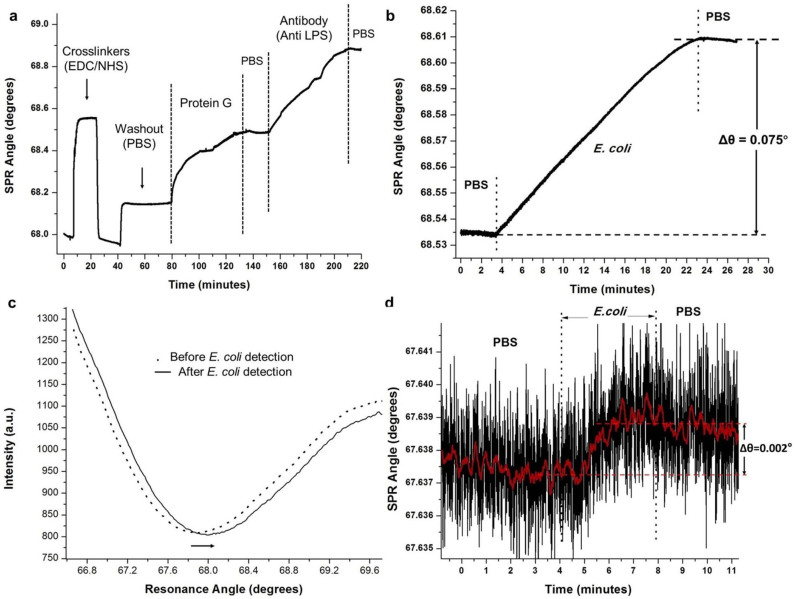
Evaluation of the platform. (a) Real time monitoring of the surface chemistry steps with respect to surface plasmon resonance angle change. The modification of the gold surfaces with crosslinkers is followed by Protein G and antibody immobilization on the surface. The signal remains constant during the wash-out steps with PBS. (b) Representative detection curve of *E. coli*. First PBS was introduced into the channel, then 100 μL of 10^6^ CFUs/mL *E. coli* was passed at 5 μL/minute, and finally PBS was introduced again. (c) Representative plot of plasmon resonance shifts after the application of *E. coli* to the biosensing platform. The arrow indicates the plasmonic shift due to the detection of GFP-expressing *E. coli*, when 10^6^ CFUs/mL *E. coli* was applied. The minimum point of the curves are the surface plasmon resonance angles. (d) Limit of detection evaluation from a 20 μL sample is shown. Protein G and anti-Lipopolysaccharide (LPS)-based surface chemistry optimized for off-device activation was used to capture and detect 10^6^ CFUs/mL *E. coli* in PBS. 20 μL of *E. coli* was passed through the channel and ~0.002° plasmon shift was observed. The 10× fluorescence images were used to count the green fluorescing spots, and we observed *E. coli* count of 32 ± 1.6 (n = 6, error is given in standard error of the mean). The red curve shows a smoothed sensogram to show the limit of detection signal.

For kinetic measurements and the evaluation of surface functionalization protocol, described in the Materials section (Functionalization of microchannels for label-free *E. coli* and *S. aureus* capture and detection section), the microchip surfaces were activated using syringe pumps at 5 μL/min flow rate, while they were mounted on the SPR platform. During the washout steps with PBS, we observed the signal stayed constant. Each modification step in the surface functionalization increased the resonance angle by changing the refractive index near the gold surface (Figure 4a). Protein G and anti-LPS binding were observed rapidly as well as other surface events at the interface (Figure 4a). Subsequently, this protocol was modified to be applied with pipettes, allowing for multiple microchip preparation in a parallel manner and used in the *E. coli* capture and detection experiments with the SPR platform. A representative resonance shift in the SPR curve upon capture and detection of bacteria is illustrated in Figure 4c.

### Limit of Detection and Standard Curves

In the literature, the limit of detection (LOD) of an SPR sensor is reported mainly as a function of the concentration of cells introduced to the system^38^. This metric does not take the sample volume into account. Therefore, we evaluated the LOD using two approaches. In the first approach, a fixed volume (100 μL) was used, and the lowest detectable bacteria concentration was evaluated. Standard curves were also provided for bacteria quantification in PBS solution and in PD fluid. In comparison, the second approach evaluates the absolute number of bacteria captured within the active area of the sensor inducing a detectable resonance shift given a fixed concentration.

**Figure 5:**
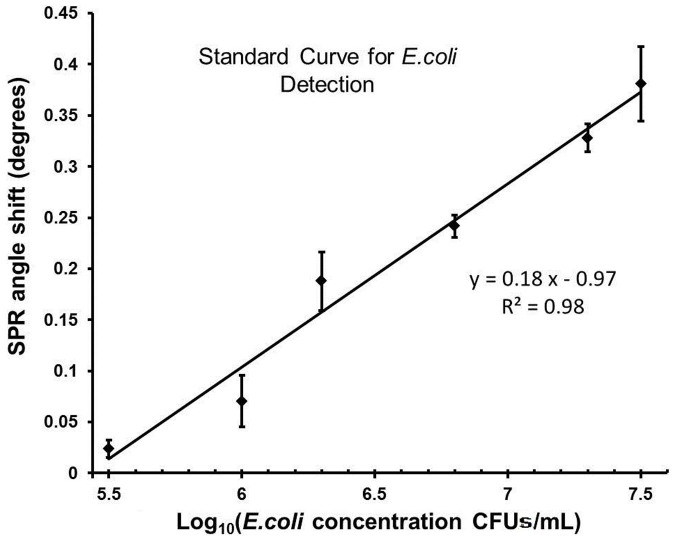
Validation with *E. coli* in PBS. The concentrations of 3.2 × 10^5^ CFUs/mL, 10^6^ CFUs/mL, 2 × 10^6^ CFUs/mL, 6.3 × 10^6^ CFUs/mL, 2 × 10^7^ CFUs/mL, and 3.2 × 10^7^ CFUs/mL were evaluated. At each concentration, 6 microchips were evaluated at 5 μL/minute for 20 minutes. The linear curve shows the least squares fit with R^2^ = 0.98 (n = 6, error bars represent standard error of the mean).

To evaluate the response range and potential applicability of the system to biological systems, we analyzed various concentrations of *E. coli* suspensions in PBS. A representative sensogram demonstrating *E. coli* detection is shown in Figure 4b. First, PBS was introduced into the surface functionalized channel, then 100 μL of 10^6^ CFUs/mL of *E. coli* was passed at 5 μL/minute, and finally PBS was introduced again to wash unbound bacteria. We observed that the signal stayed constant during PBS runs, whereas a signal increase was observed in time when the *E. coli* was introduced. For the response curve analysis, *E. coli* concentrations of 3.2 × 10^5^ CFUs/mL, 10^6^ CFUs/mL, 2 × 10^6^ CFUs/mL, 6.3 × 10^6^ CFUs/mL, 2 × 10^7^ CFUs/mL, and 3.2 × 10^7^ CFUs/mL spiked in PBS were evaluated at 5 μL/minute flow rate (Figure 5). At each concentration, six microchips were run for 20 minutes to generate reliable signal levels. The platform demonstrated a linear response (R^2^ = 0.98) between the plasmon signal and the logarithm of the *E. coli* concentration within the 3.2 × 10^5^–3.2 × 10^7^ CFUs/mL range. We observed plasmon shifts of ~0.02° and ~0.38° at concentrations 3.2 × 10^5^ CFUs/mL and 3.2 × 10^7^ CFUs/mL, respectively. The standard curve shown in Figure 5 can be used to calculate an unknown *E. coli* concentration spiked in PBS for quantitative analysis. The LOD for *E. coli* capture and detection with direct immunoassays in SPR-based systems is ~10^6^ CFUs/mL^39^,^40^. Lower LODs have also been reported, but with bulky SPR systems that employ sandwich assays to improve the LOD^41^. For instance, a flow-cell based SPR sensor was reported to detect *Salmonella typhimurium* at 2.5 × 10^5^ cells/mL and *Salmonella* *enteritidis* at 2.5 × 10^8^ cells/mL with a sandwich assay^42^. It has been also noted that sample preparation may influence the signal levels. For instance, *E. coli* was detected using a sandwich assay with an LOD of 10^4^ CFUs/mL when the bacteria was lysed, 10^5^ CFUs/mL when the bacteria was heat-killed and with 10^6^ CFUs/mL with untreated *E.coli*^43^. The portable microfluidic-based SPR platform reported here provides a comparable with these different platforms. Although sandwich assays, sample labeling and modified sample preparation techniques can improve the reported LOD, these preprocesses increase the complexity, thus reducing the applicability in POC settings^44^. There are existing commercial SPR platforms reported^45^,^46^,^47^,^48^,^49^,^50^,^51^ (Table S1). For instance, Biacore device is notably reported to detect 25 CFUs/mL of *E. coli*, using 25 μL samples, corresponding to an absolute value of 0.62 CFU/mL of *E.coli* (less than 1 CFU) in the total evaluated sample volume. This performance is reached at the expense of portability, since the Biacore laboratory equipment weighing 50 kg is not suitable for POC applications^44^,^52^. In comparison, this work reports a portable device (0.85 kg) suitable for POC applications, with a favorable LOD in comparison to the mean of the values given in Table S1. The platform reported here requires lower sample volume, as well as provides significant advantages using disposable chips and portable reader for POC applications.

**Figure 6:**
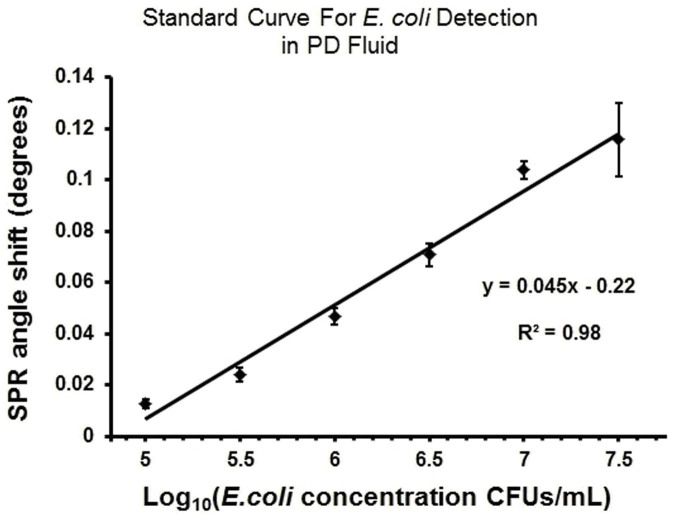
Validation with *E. coli* spiked in peritoneal dialysis (PD) fluid. The concentration range of 10^5^ CFUs/mL to 3.2 × 10^7^ CFUs/mL was evaluated. At each concentration, 6 microchips were evaluated at 5 μL/minute for 20 minutes. The linear curve shows the least squares fit with R^2^ = 0.98 (n = 6, error bars represent standard error of the mean).

As an example of a biologically relevant fluid, we evaluated the detection of *E. coli* samples spiked in PD fluid at the concentration range of 10^5^ CFUs/mL to 3.2 × 10^7^ CFUs/mL (Figure 6). The platform performed well in comparison to the PBS experiments, with an R^2^ value of 0.98 for the linear fit. At the highest concentration of 3.2 × 10^7^ CFUs/mL we observed a signal of ~0.11 degrees in comparison to the ~0.38 degrees of PBS experiments given in Figure 5. We observed the slope of the linear fit was slightly smaller. PBS and PD fluid have different chemical and optical properties (*e.g.*, refractive index, pH), which may contribute to the resonance angle shifts observed in experiments at the same *E. coli* concentrations. During the PD fluid experiments the experimental parameters (*i.e*., surface functionalization, chip design) were kept the same as in the PBS experiments, however a different batch of gold coated chips were used, which may also have contributed to the difference in the observed signal levels.

In the second LOD approach, we evaluated the absolute number of cells that the system is sensitive to, which was defined as the absolute LOD. Multiple sample volumes between 20 μL and 100 μL were evaluated to characterize the experimental detection limitation of the system. The active area on the gold chip surface (5.3 × 10^6^ μm^2^) inducing this signal was selected (Figure S2) and fluorescence images at 10× magnification were collected after separating the gold substrates from the microchips. For each chip, nine images were acquired under 10× magnification, and the GFP-expressing bacteria were counted inducing the signal. From an engineering point-of-view, we performed experiments at increasing sample volumes to evaluate detection limitations. Specifically, 10^6^ CFUs/mL of *E. coli* in 20 μL PBS solution were applied to five chips for 4 minutes, and the sensor recorded a ~0.002° shift in the resonance angle when chips were run at 5 μL/min (Figure 4d). The ~0.002° shift was found to result from 32 ± 1.6 CFUs/mL of *E. coli* (n = 5, and error is given in standard error of the mean). The instrument resolution (~0.002°) reported in the calibration section is commensurate with the experimentally measured limitation (~0.002°) at 20 μL (see Calibration section under Materials and Methods). Further, 10^6^ CFUs/mL of *E. coli* spiked in 30 μL PBS solution were applied to three other chips for 6 minutes, and a 0.004° shift response was observed. Nine images at 10× magnification were used to count the bacteria, and it was found that the ~0.004° plasmon shift was induced by 55 ± 2.1 CFUs/mL of *E. coli* captured within the active area (Figure S3). Here, a higher number of chips were run at increasing volumes, and the system responses were evaluated. We carefully characterized the platform and chose the most robust and reliable operating conditions. By considering platform performance and clinical applicability, 100 μL sample volume was used in our experiments with *E. coli*. Since the measured signal levels in Figures 4, 5 and 6 were on the order of 0.1°–0.3° for *E. coli*, and are two orders of magnitude higher than the experimental instrument resolution (~0.002°), the chosen volume of 100 μL regularly provided reliable and robust conditions. The LOD can potentially be improved by incorporating on-chip or off-chip microfluidic functional elements to increase the capture efficiency. A good example of this would be utilizing the same fluid volume multiple times to increase the captured bacteria.

### Specificity and Multiplexing

**Figure 7:**
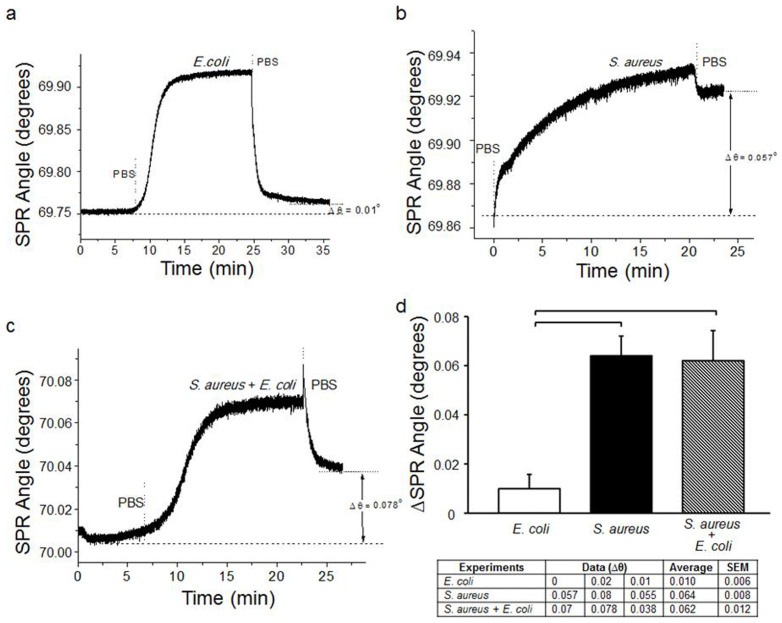
Specificity, selectivity and multiplexing of the SPR platform. SPR chips were decorated with anti-LTA antibodies, which are only specific to *S. aureus* (Gram + bacteria). (a) *E. coli* spiked in PBS (5 × 10^6^ CFUs/mL) were applied onto anti-LTA antibody modified surfaces. (b) *S. aureus* spiked in PBS (5 × 10^6^ CFUs/mL) were applied onto anti-LTA antibody modified surfaces. (c) For specificity and selectivity experiments, *S. aureus* and *E. coli* were mixed in PBS at the concentrations reported above. The mixture was then applied onto anti-LTA antibody modified surfaces. (d) Changes in SPR angle were recorded for each case. For statistical analysis, one-way analysis of variance (ANOVA) with Tukey's posthoc test was performed with Bonferroni's Multiple Comparison Test for equal variances for multiple comparisons. Statistical significance threshold was set at 0.05 (n = 3, p < 0.05), and brackets represented statistical significant differences between groups. Error bars represented mean ± standard errors of the mean (SEM).

The specificity and selectivity of the proposed platform was evaluated with three sets of experiments. The first experiment was conducted by eluting *E. coli* over anti-LTA antibody-modified microfluidic chips. We observed that *E. coli* binding is minimum since anti-LTA antibody is specific for Gram + bacteria, which exhibited no affinity to *E. coli* (Figure 7a). As a result, there was minimal cross-reactivity (nonspecific binding) observed against *E. coli* on anti-LTA antibody-modified surfaces (Δθ = 0.01°) (Figure 7a). The second experiment was designed by elution of *S. aureus* over anti-LTA modified surface. Figure 7b shows the typical binding curve of *S. aureus* on anti-LTA-modified surface, which yielded 0.057° shift in SPR angle. Further, as a third experiment (negative control), we examined a mixture of *S. aureus* and *E. coli* on anti-LTA antibody-modified surfaces (Figure 7c). Statistical analysis demonstrated that there is no significant difference between the results obtained from *S. aureus* and mixture cases (n = 3, p > 0.05) (Figure 7d). Since *E. coli* has no significant interactions to the anti-LTA antibody-modified surface (Figure 7a), the SPR angle shift originated due to *S. aureus* binding to the surfaces, confirming selective capture of *S. aureus* from the mixture. Additionally, the SPR angle change in *E. coli* experiments on the anti-LTA antibody-modified surfaces were observed to be statistically different than *S. aureus* and mixture experiments (n = 3, p < 0.05). Thus, we demonstrated the specificity, selectivity and multiplexing capability of our platform.

The development of POC platforms is crucial both for field-based diagnostics and personalized medicine applications^53^,^54^. However, requirements for the portability of POC instruments hinder the transition of these devices to diagnostic and monitoring applications at the bed-side, primary care and resource-constrained settings. The presented platform incorporates a hand-held optical reader, which utilizes disposable microchips adaptable to portable and versatile POC applications. The system operates with small sample volumes (100 μL), gives results within 20 minutes, and was made from inexpensive equipment. The portability of the device (0.85 kg) allows simple transportation without compromising sensitivity. The microchips can be disposed of without raising any contamination issues. The chip fabrication and surface activation procedures are high throughput, and the whole process can be automated in the future. Further, plasmonic-based technologies are label-free and detect the target directly with less complicated protocols compared to fluorescent techniques^55^. Plasmonics allow detection of ultralow concentrations of bioagents^24^,^56^,^57^. Therefore, merging microfluidics with plasmonic based technologies is enabling new operating modalities. For instance, recent developments in SPR imaging (SPRi) allowed high throughput on-chip sensors^58^ and on-chip immunoassays^59^. LSPR based platforms are also merging with microfluidics^60^. Development of such novel plasmonic-based microfluidic sensors will potentially contribute to infectious disease diagnosis and monitoring both at the POC and primary care settings.

## Conclusions

We have developed a portable, label-free pathogen detection platform that merged microfluidic and SPR technologies on a single platform and demonstrated detection and quantification of bacterial pathogens. Using a plasmonic-based microchip sensitive pathogen detection is attained, which can potentially be used for POC applications. We further evaluated the response of the platform with detection of *E. coli-spiked* in PBS and PD fluid. The demonstrated system can potentially be generalized to other pathogens or for immunodiagnostics, given that there are well-defined biomarkers for the targeted applications. For instance, the presented platform is potentially applicable to other bacterial and viral diseases such as influenza, hepatitis, AIDS, and tuberculosis. Therefore, with the use of disposable, easy-to-fabricate and sensitive plasmonic surfaces with a specific surface chemistry on a label-free microfluidic platform, we address some of the major problems of current biosensing tools at the POC (*i.e*., portability, cost, small sample size and practical operation requirements).

## Author Contributions

O.T., A.D. and U.D. developed the idea; A.D. and U.D. collaborated on the paper; O.T., A.D. and U.D. designed the experimental approach; O.T., U.H.Y., F.I., N.G.D., O.O.E., B.T., C.C., S.R., K.S., N.N. and H.S. performed the experiments; O.T., U.H.Y., F.I. and U.D. analyzed the data; O.T., U.H.Y., F.I., A.D. and U.D. wrote the manuscript. All authors reviewed the manuscript.

## Supplementary Material

Supplementary Information Supplementary Information
