# Supplementary material for: Portable Microfluidic Integrated Plasmonic Platform for Pathogen Detection
Source: Sci Rep. 2015 Mar 24;5:9152. doi: 10.1038/srep09152 (PMC4371189; doi:10.1038/srep09152)
Supplement: Supplementary Information [file srep09152-s1.doc]

SUPPORTING INFORMATION

Portable Microfluidic Integrated Plasmonic Platform for Pathogen Detection

Onur Tokel,*^a,#^* Umit Hakan Yildiz,*^b,#^* Fatih Inci,*^b^*  Naside Gozde Durmus,^c,d^ Okan Oner Ekiz,*^e^* Burak Turker, *^e^* Can Cetin, *^a^* Shruthi Rao, *^a^* Kaushik Sridhar, *^a^* Nalini Natarajan, *^a^* Hadi Shafiee, *^a^* Aykutlu Dana,*^e,*^* and Utkan Demirci *^a,b, *^*

^a^ Demirci Bio-Acoustic-MEMS in Medicine (BAMM) Laboratory, Center for Biomedical Engineering, Department of Medicine, Brigham and Women’s Hospital, Harvard Medical School, Boston, MA, USA.

^b^ Demirci Bio-Acoustic-MEMS in Medicine (BAMM) Laboratory, Stanford University School of Medicine, Canary Center at Stanford for Cancer Early Detection, Palo Alto, CA, USA.

^c^ Department of Biochemistry, Stanford School of Medicine, Stanford, CA, USA.

^d^ Stanford Genome Technology Center, Stanford University, Palo Alto, CA, USA.

^e^ UNAM Institute of Materials Science and Nanotechnology, Bilkent University, 06800 Ankara, Turkey.

*^#^ The authors contributed equally.*

*^*^Corresponding authors:*

*Utkan Demirci, PhD*

Tel: +1-650-906-9227

E-mail: utkan@stanford.edu

*Aykutlu Dana, PhD*

Tel: + 90-536-300 6515

E-mail: aykutlu@fen.bilkent.edu.tr

***Table S1.*** *Existing SPR technology for E. coli detection.*

| **Bacteria** | **SPR brand** | **Assay Type** | **LOD (CFUs/mL)** | **Sample**  **Volume** | **CFU in volume** | ***Ref*** |
| --- | --- | --- | --- | --- | --- | --- |
| *E. coli* | Spreeta | Direct | 10^2^-10^3^ | 2 mL | ~1500 | [^1^](#_ENREF_1) |
| *E. coli* | Reichert | Direct/Sandwich/Direct | 10^6^ /10^3^ /10^4^ | 0.5 mL | 5x10^5^/500/5000 | [^2^](#_ENREF_2) |
| *E. coli* | Biacore | Direct | 25 | 25 μL | 0.6 | [^3^](#_ENREF_3) |
| *E. coli* | Spreeta | Direct | 90 | 1 mL | 90 | [^4^](#_ENREF_4) |
| *E. coli* | Custom-built | Direct | 1.4 x 10^4^ | 1 mL | 1.4 x 10^4^ | [^5^](#_ENREF_5) |
| *E. coli* | Multiskop | Direct | 10^4^ | N/A | N/A | [^6^](#_ENREF_6) |
| *E. coli* | Biosuplar | Direct | 10^7^ | 200 μL | 2x10^6^ | [^7^](#_ENREF_7) |
| *E. coli* | **This work** | Direct | 10^5^ | 100 μL | 10^4^ | This work |

**
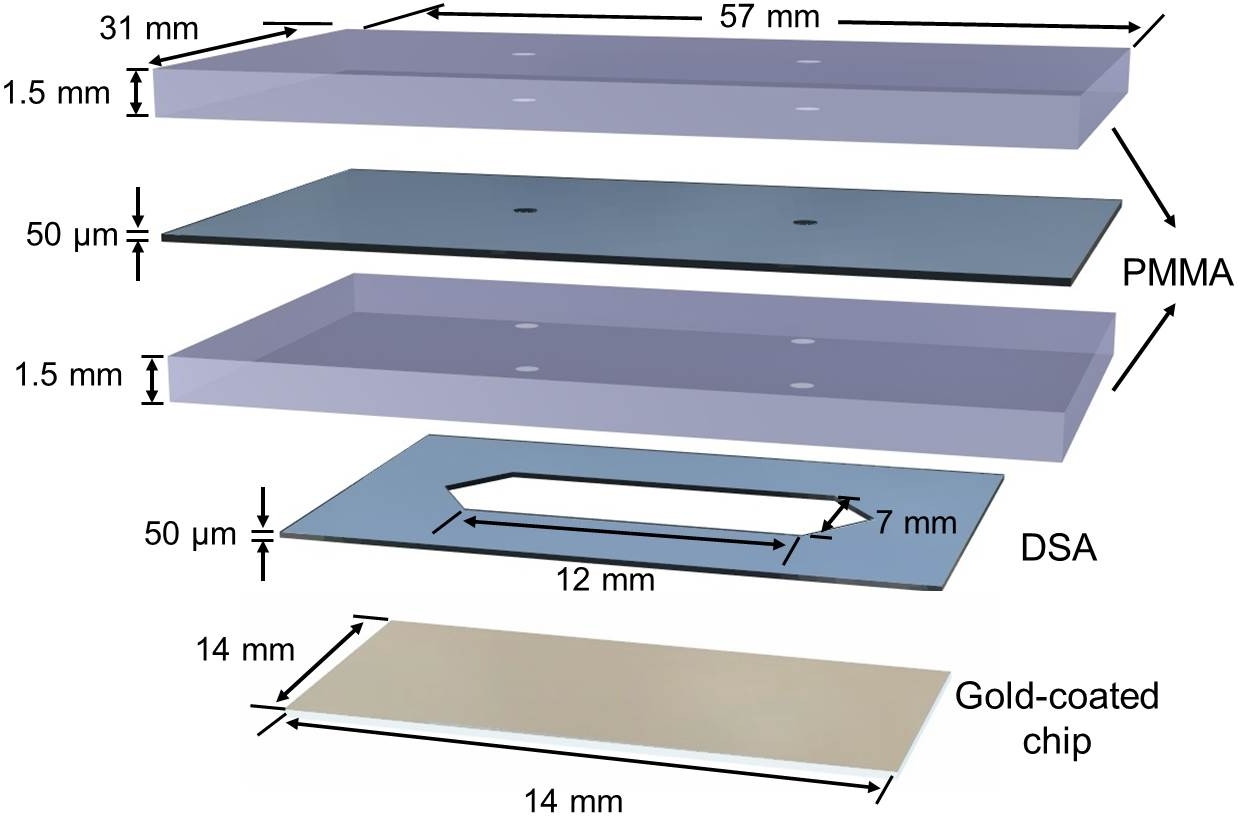
**

**Figure S1:** Schematic of the microchip for the SPR platform. Two layers of PMMA and two layers of DSA were cut by a laser cutter. In the assembled microchip, a DSA layer formed a microchannel between the base PMMA and the gold coated chip. The microchannel dimentions were 12mm x 7 mm x 50 μm (length x width x height). The PMMA layers were 3 mm thick and the glass substrate was 0.5 mm thick. The assembled chip thickness was about 6.5 mm. The gold surface was functionalized with antibodies specific to *E. coli* capture. Two openings on the PMMA (0.72 mm diameter) formed the inlet and outlet ports for fluid transport during capture and detection experiments.


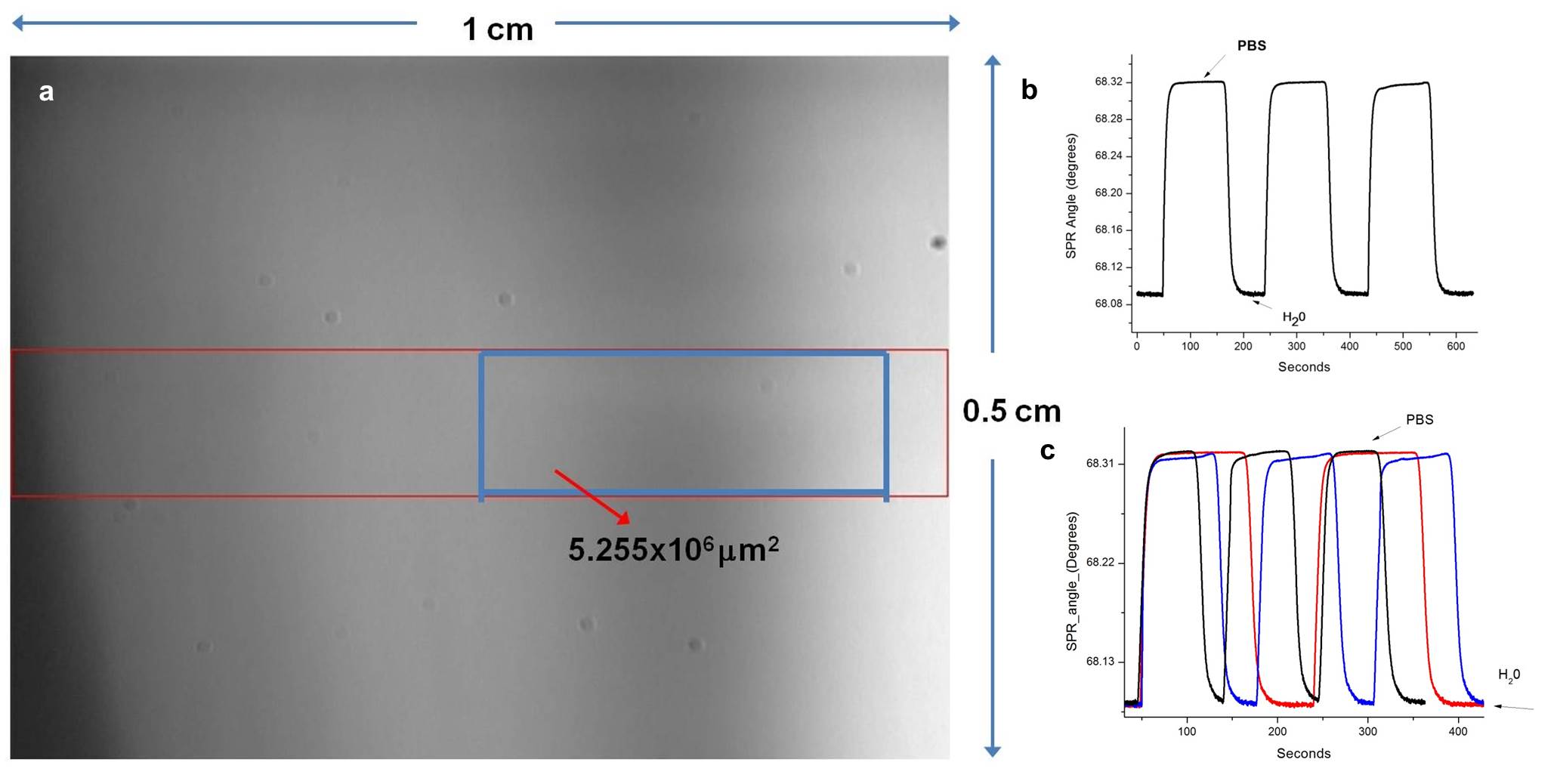


**Figure S2: (a)** The field of view of the CMOS sensor corresponded to an area of 1 cm x 0.5 cm on the gold chip surface. The resonance condition was satisfied at certain angles corresponding to a band which was observed as a dark shadow on the camera. The custom software averaged the pixel intensities in the chosen area (red marked rectangle) and calculated the resonance angle from the curve. The resonance angle was also monitored as the minimum of the curve shown in Figure 4c. Limit of detection measurements were performed in the area marked with the blue rectangle. **(b)** A two inlet microchip was prepared for response time measurements. Distilled water and PBS were alternately applied to the platform. The switching of the liquids was performed by syringe pumps and 5 μL/min flow speed was used. The rise time (time to increase from 10% to 90% of the difference between the water and PBS resonances) of the switching was observed as 8 seconds. **(c)** This value was checked at multiple switching periods and it remained constant.

**
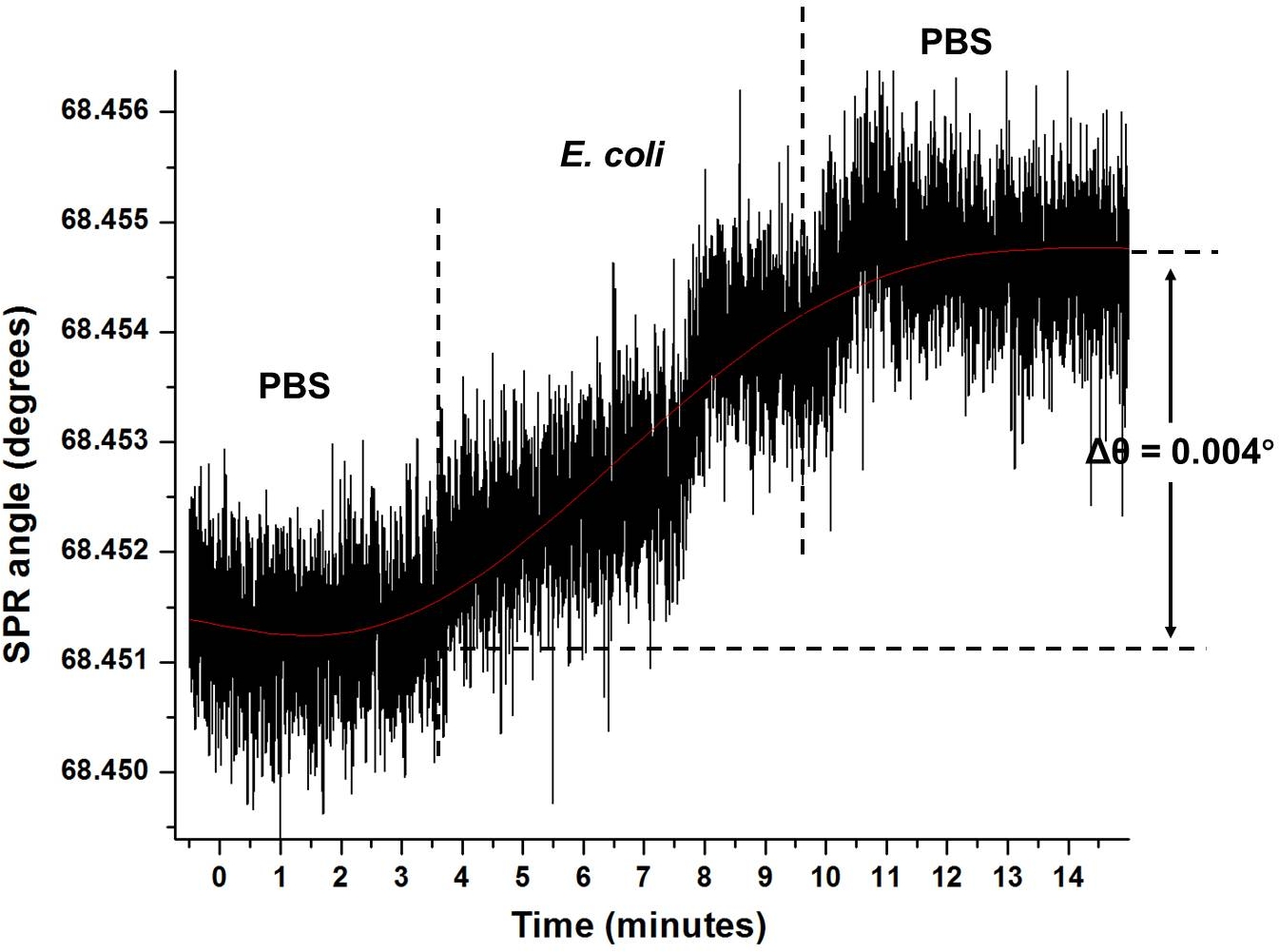
**

**Figure S3:** Limit of detection evaluation with a 30 μL sample. A Protein G and anti-LPS based surface chemistry was utilized to capture and detect 10^6^ CFUs/mL *E. coli* in PBS*.* 30 μL of *E. coli* was passed through the channel and ~0.004° plasmon shift was observed. The 10X fluorescence images taken were used to count the green fluorescing spots to give an *E. coli* count of 55 ± 2.1 (n=3, error is given in standard error of the mean) causing the observed shift. The red curve shows a polynomial fit to the sensogram.

**REFERENCES**

1. Waswa, J., Irudayaraj, J. & DebRoy, C. Direct detection of E. Coli O157:H7 in selected food systems by a surface plasmon resonance biosensor. *LWT - Food Sci. Technol.* **40**, 187-192; DOI: 10.1016/j.lwt.2005.11.001 (2007).

2. Subramanian, A., Irudayaraj, J. & Ryan, T. A mixed self-assembled monolayer-based surface plasmon immunosensor for detection of E. coli O157:H7. *Biosens. Bioelectron.* **21**, 998-1006; DOI:10.1016/j.bios.2005.03.007 (2006).

3. Waswa, J. W., Debroy, C. & Irudayaraj, J. Rapid detection of salmonella enteritidis and escherichia coli using surface plasmon resonance biosensor. *J. Food Process Eng.* **29**, 373-385; DOI:10.1111/j.1745-4530.2006.00071.x (2006).

4. Dudak, F. C. & Boyacı, İ. H. Development of an immunosensor based on surface plasmon resonance for enumeration of Escherichia coli in water samples. *Food Res. Int.* **40**, 803-807; DOI: 10.1016/j.foodres.2007.01.011 (2007).

5. Taylor, A. D. *et al.* Quantitative and simultaneous detection of four foodborne bacterial pathogens with a multi-channel SPR sensor. *Biosens. Bioelectron.* **22**, 752-758; DOI: 10.1016/j.bios.2006.03.012 (2006).

6. Oh, B. K., Kim, Y. K., Bae, Y. M., Lee, W. H. & Choi, J. W. Detection of Escherichia coli O157 : H7 using immunosensor based on surface plasmon resonance. *J Microbiol. Biotechn.* **12**, 780-786 (2002).

7. Maalouf, R. *et al.* Label-Free Detection of Bacteria by Electrochemical Impedance Spectroscopy:  Comparison to Surface Plasmon Resonance. *Anal. Chem.* **79**, 4879-4886; DOI:10.1021/ac070085n (2007).
